# Comparison of Long-Term Survival Outcomes of T4a and T4b Colorectal Cancer

**DOI:** 10.3389/fonc.2021.780684

**Published:** 2022-01-06

**Authors:** Ji Ha Lim, Jung Wook Huh, Woo Yong Lee, Seong Hyeon Yun, Hee Cheol Kim, Yong Beom Cho, Yoon Ah Park, Jung Kyong Shin

**Affiliations:** Department of Surgery, Samsung Medical Center, Sungkyunkwan University School of Medicine, Seoul, South Korea

**Keywords:** AJCC guideline, colorectal cancer, serosal exposure, organ invasion, survival

## Abstract

**Background:**

Although T4b is known to have worse oncologic outcomes, it is unclear whether it truly shows a worse prognosis. This study aims to compare the survival differences between T4a and T4b.

**Methods:**

Patients who were pathologically diagnosed with T3 and T4 colorectal adenocarcinoma from 2010 to 2014 were included (T3, n = 1822; T4a, n = 424; T4b, n = 67). Overall survival (OS) and cancer-specific survival (CSS) were compared between T4a and T4b using the Kaplan-Meier method and log-rank test.

**Results:**

In stage II, T4a had better OS and CSS than T4b (5-year OS, 89.5% vs. 72.6%; 5-year CSS, 94.4% vs. 81.7%, all p < 0.05), however, in stage III, there were no significant differences in survivals between groups (all p > 0.05). In multivariable analysis, T classification was not an independent risk factor for OS (p > 0.05). However, for CSS, when respectively compared to T3, T4b (HR 3.53, p < 0.001) showed a relatively higher hazard ratio than T4a (HR 2.27, p < 0.001).

**Conclusions:**

T4a showed more favorable OS and CSS than T4b, especially in stage II. Our findings support the current AJCC guidelines, in which T4b is presented as a more advanced stage than T4a.

## Introduction

Colorectal cancer (CRC) is one of the most common types of cancer. In 2017, it was the second most common malignancy, following gastric cancer, and had the third-highest cancer mortality rate in South Korea (1). The incidence of CRC remains high compared to that of other cancers. To allow doctors to design treatment plans and determine a prognosis for each patient, patients diagnosed with CRC are often classified according to the American Joint Committee on Cancer (AJCC)’s tumor-node-metastasis (TNM) staging system.

In the 7th edition of the AJCC guidelines on colorectal cancer, stage T4 was divided into T4a and T4b (2). T4b may be considered more severe due to other organs/structural invasion, and several studies confirmed that T4b showed worse outcomes than T4a (3–5). However, T4a tumor that invades the free serosa may have a greater chance of peritoneal seeding (6–8), and it is still unclear and controversial whether T4a tumors are truly associated with a better prognosis than T4b tumors (6–10). The purpose of this study is to compare the long-term survival outcomes of patients with T4a and T4b tumors and determine if there are any differences in the outcomes.

## Materials and Methods

The records of patients who were first diagnosed with colorectal adenocarcinoma and underwent surgery in Samsung Medical Center (SMC) from January 2010 to December 2014 were collected for this study. Pathologically confirmed T3 and T4 patients who underwent curative-intent surgery with R0 resection were included. Patients with (1) stage IV disease, (2) recurrent colorectal cancer, (3) hereditary colorectal diseases, (4) no records of preoperative carcinoembryonic antigen (CEA)/carbohydrate antigen (CA) 19-9, (5) neoadjuvant chemotherapy with or without radiotherapy, (6) other organ malignancies and (7) mid to lower rectal cancer were excluded. As a result, a total of 1822 patients were analyzed. We obtained approval from the Institutional Review Board of Samsung Medical Center (SMC 2021-01-070).

Collected baseline characteristics included age, sex, body mass index (BMI), American Society of Anesthesiologists (ASA) score, preoperative CEA, and CA 19-9 level. All tissue samples taken after colectomy or proctectomy were evaluated and confirmed by several pathologists based on AJCC guidelines (8th edition) (11). The definitions of T3, T4a, and T4b in the guidelines are as follows: T3, tumor invades through the muscularis propria into peri-colorectal tissues; T4a, tumor penetrates the surface of the visceral peritoneum; T4b, tumor directly invades or is adherent to other organs or structures (2, 9). We also obtained data on tumor size; pathologic tumor-nodal stage; lymphatic, vascular, and perineural invasion; tumor budding; and microsatellite instability (MSI) status for all patients. Each patient underwent colectomy or proctectomy according to the location of the tumor (12–14).

If tumor invasion or adhesion to other organs were found, en-bloc resection was conducted. The right colon included the cecum to the mid-transverse colon, and the left colon included the distal transverse colon to the rectosigmoid colon. Postoperative complications were classified by the Clavien-Dindo classification (CDC) system (15, 16). Patients who had complications of grade III, which requires surgical, endoscopic, or radiological intervention, and higher were investigated. Readmission was defined as admission again within 30 days after discharge.

Adjuvant therapy was done according to the National Comprehensive Cancer Network (NCCN) guidelines. In patients who refused recommended chemotherapy, routine follow-up was performed as in all other non-chemotherapy-treated patients. Patients were first followed up within two weeks after discharge, then every 3 months for 2 years, and then every 6 months until 5 years after surgery. After that, the further follow-up period was determined at the discretion of the surgeons. Follow-up examination included serum CEA level, chest CT, AP-CT or MRI, colonoscopy, and EGD or PET-CT, if recurrence was suspected.

The primary outcome was long-term survival outcome and the secondary outcome was pathologic differences between T4a and T4b tumors.

All statistical analyses were performed using SAS version 9.4 (SAS Institute Inc, Cary, NC, USA) and SPSS (version 27.0, SPSS Inc., Chicago, IL). A p-value of <0.05 was considered statistically significant. Comparisons among the T3, T4a, and T4b groups were performed using χ2 or Fisher’s exact test as needed for categorical variables, while the Kruskal-Wallis test was used for continuous variables. *Post-hoc* analysis was performed for variables with a p-value of less than 0.05 among the three groups. The survival rates and curves were expressed using the Kaplan-Meier method and compared using log-rank tests. Cox regression analysis of overall survival (OS) and cancer-specific survival (CSS) was conducted to identify the factors that have an influence on survivals. After screening for significant variables in univariable regression, it was estimated through variable selection using backward elimination method in multivariable Cox regression. Multicollinearity was reviewed as a variance inflation factor (VIF), and variables with abnormally large VIFs were excluded from the final model.

## Results

A total of 1822 patients who underwent surgery for colorectal cancer from 2010 to 2014 and who were pathologically confirmed to have T3 and T4 colorectal cancer were included in the study (T3, n = 1822; T4a, n = 424; T4b, n = 67).

A comparison of baseline characteristics among the T3, T4a, and T4b groups is presented in **Table 1**. There were no statistically significant differences in age, sex, ASA score, or the number of tumors among the three groups. Following *post-hoc* analysis, the proportions of patients who showed elevated preoperative CEA levels and cancer obstruction were not significantly different between the T4a and T4b groups. More T4b than T4a patients showed elevated preoperative CA 19-9 levels (31.3% vs. 17.0%, respectively; *post-hoc* p = 0.026). Moreover, more T4b patients presented with cancer perforation than T4a patients (10.5% vs. 2.8%, respectively; *post-hoc* p = 0.023), and the mean tumor size was larger in T4b than T4a patients (8.07 ± 2.89 cm vs. 4.95 ± 3.00 cm, *post-hoc* p < 0.001). More T4b cases were emergent and open cases (emergent operation, 11.9% vs. 3.3%, p = 0.013; open surgery, 68.6% vs. 17.7%, p < 0.001). T4b patients also showed a longer mean operation time (223.03 ± 107.53 min vs. 159.40 ± 62.74 min, *post-hoc* p < 0.001). T4a patients showed the same proportion of right and left colon involvement (37% each), but T4b was more often seen on the right side (right colon, 47.8%; left colon, 22.4%) (*post-hoc* p = 0.018) (**Table 1**).

**Table 1:** Baseline characteristics of the study subjects.

Parameters	Total (n = 1822)	T3 (n = 1331)	T4a (n = 424)	T4b (n = 67)	p-value	*post hoc*
						p-value
**Age, mean ± SD, years**	60.73 ± 11.67	60.64 ± 11.51	61.10 ± 11.99	60.39 ± 12.91		
<60year	824 (45.2%)	606 (45.5%)	188 (44.3%)	30 (44.8%)	0.910	
≥60year	998 (54.8%)	725 (54.5%)	236 (55.7%)	37 (55.2%)		
**Sex**						
Male	1049 (57.6%)	775 (58.2%)	243 (57.3%)	31 (46.3%)	0.153	
Female	773 (42.4%)	556 (41.8%)	181 (42.7%)	36 (53.7%)		
**BMI, median (range), kg/m2**	23.5 (21.6-25.6)	23.8(21.8-25.8)	23.0(21.0-25.0)	21.0(20.0-24.0)	<0.001	0.003
**ASA score**						
1	651 (35.7%)	478 (35.9%)	153 (36.1%)	20 (29.9%)	0.114	
2	1092 (59.9%)	802 (60.3%)	244 (57.6%)	46 (68.7%)		
3	79 (4.4%)	51 (3.8%)	27 (6.3%)	1 (1.4%)		
**Preoperative CEA**						0.107
≥5 ng/ml	506 (27.8%)	338 (25.4%)	137 (32.3%)	31 (46.3%)	<0.001	
<5 ng/ml	1316 (72.2%)	993 (74.6%)	287 (67.7%)	36 (53.7%)		
**Preoperative CA19-9**					0.021	0.026
≥37 ng/ml	347 (19.1%)	254 (19.1%)	72 (17.0%)	21 (31.3%)		
<37 ng/ml	1475 (80.9%)	1077 (80.9%)	352 (83.0%)	46 (68.7%)		
**Cancer obstruction**						
Yes	414 (22.7%)	234 (17.6%)	145 (34.2%)	35 (52.2%)	<0.001	0.201
No	1408 (77.3%)	1097 (82.4%)	279 (65.8%)	32 (47.8%)		
**Cancer Perforation**						
Yes	19 (1.0%)	0 (0%)	12 (2.8%)	7 (10.5%)	<0.001	0.023
No	1803 (99.0%)	1331 (100%)	412 (97.2%)	60 (89.5%)		
**Tumor location**						
Right colon	527 (28.9%)	338 (25.4%)	157 (37.0%)	32 (47.8%)	<0.001	0.018
Left colon	687 (37.7%)	515 (38.7%)	157 (37.0%)	15 (22.4%)		
Rectum	608 (33.4%)	478 (35.9%)	110 (26.0%)	20 (29.8%)		
**Tumor size, mean ± SD, cm**	5.12 ± 2.41	5.02 ± 2.05	4.95 ± 3.00	8.07 ± 2.89	<0.001	<0.001
**Type of surgery**						
Elective	1784 (97.9%)	1315 (98.8%)	410 (96.7%)	59 (88.1%)	<0.001	0.013
Emergent	38 (2.1%)	16 (1.2%)	14 (3.3%)	8 (11.9%)		
**Operative technique**						
Open	346 (19.0%)	225 (16.9%)	75 (17.7%)	46 (68.6%)	<0.001	<0.001
MIS^a)^	1476 (81.0%)	1106 (83.1%)	349 (82.3%)	21 (31.4%)		
**Operative time, mean ± SD, min**	157.99 ± 56.83	154.26 ± 48.66	159.40 ± 62.74	223.03 ± 107.53	<0.001	<0.001

SD, standard deviation; BMI, body mass index; ASA, American Society of Anesthesiologists; CEA, carcinoembryonic antigen; CA 19-9, Carbohydrate antigen 19-9; MIS, minimally invasive surgery.

^a)^This includes hand-assisted laparoscopy, total laparoscopy, and robotic surgery.

Regarding pathologic outcomes, all investigated pathologic variables showed statistically significant differences among T3, T4a, and T4b patients; however, in the *post-hoc* analysis, only nodal status, number of positive lymph nodes, and lymphatic and perineural invasion showed significantly differed between the T4a and T4b groups. T4a patients showed higher nodal status (N1, 32.8% vs. 43.6%; N2, 19.4% vs. 30.2%, *post-hoc* p < 0.001) and a greater mean number of positive lymph nodes (2.00 ± 3.41 vs. 2.71 ± 3.03, *post-hoc* p = 0.044). Lymphatic and perineural invasion were also more common in T4a than T4b (lymphatic invasion, 38.8% vs. 59.0%, *post-hoc* p = 0.001; perineural invasion, 34.3% vs. 52.4%, *post-hoc* p = 0.003). MSI-high (MSI-H) colorectal cancer patients were more in T4b groups (5.2% vs. 17.9%, *post-hoc* p < 0.001). In postoperative outcome, Clavien-Dindo classification (CDC) grade 3 or higher postoperative complications were more common in T4b patients than in T4a patients (11.9% vs. 3.3%, *post-hoc* p = 0.013). Adjuvant chemotherapy was performed in 73.1% of all cases (T3, 67.7%; T4a, 89.2%; T4b, 79.1%) and the chemotherapy completion rate was 91.6% (T3, 92.0%; T4a, 90.5%; T4b, 92.5%). There were no significant differences in the rates of readmission, recurrence, or mortality between the T4a and T4b groups (all p > 0.05) (**Table 2**).

**Table 2:** Postoperative outcomes.

Parameters	Total (n = 1822)	T3 (n = 1331)	T4a (n = 424)	T4b (n = 67)	p-value	*post hoc*
						p-value
**Cell differentiation**						
WD	334 (18.3%)	285 (21.4%)	44 (10.4%)	5 (7.5%)	<0.001	0.792
MD	1384 (76.0%)	990 (74.4%)	342 (80.7%)	52 (77.6%)		
PD	104 (5.7%)	56 (4.2%)	38 (8.9%)	10 (14.9%)		
**Nodal status**						
N0	790 (43.4%)	647 (48.6%)	111 (26.2%)	32 (47.8%)	<0.001	<0.001
N1	676 (37.1%)	469 (35.2%)	185 (43.6%)	22 (32.8%)		
N2	356 (19.5%)	215 (16.2%)	128 (30.2%)	13 (19.4%)		
**Positive lymph node, mean ± SD, n**	1.90 ± 2.85	1.63 ± 2.71	2.71 ± 3.03	2.00 ± 3.41	<0.001	0.044
**Lymphatic invasion**						
Yes	733 (40.2%)	457 (34.3%)	250 (59.0%)	26 (38.8%)	<0.001	0.001
No	1089 (59.8%)	874 (65.7%)	174 (41.0%)	41 (61.2%)		
**Perineural invasion**						
Yes	580 (31.8%)	335 (25.2%)	222 (52.4%)	23 (34.3%)	<0.001	0.003
No	1242 (68.2%)	996 (74.8%)	202 (47.6%)	44 (65.7%)		
**Vascular invasion**						
Yes	342 (18.8%)	221 (16.6%)	110 (25.9%)	11 (16.4%)	<0.001	0.378
No	1480 (81.2%)	1110 (83.4%)	314 (74.1%)	56 (83.6%)		
**Tumor Budding**						
Positive	1095 (60.1%)	717 (53.9%)	331 (78.1%)	47 (70.2%)	<0.001	0.609
Negative	727 (39.9%)	14 (46.1%)	93 (21.9%)	20 (29.8%)		
**MSI-H**						
Yes	115 (6.3%)	81 (6.1%)	22 (5.2%)	12 (17.9%)	<0.001	<0.001
No	1684 (92.4%)	1238 (93.0%)	396 (93.4%)	50 (74.6%)		
**Postoperative complication** **(CDC≥grade 3)**	79 (4.3%)	57 (4.3%)	14 (3.3%)	8 (11.9%)	0.013	0.013
**Length of stay, mean ± SD, day**	8.74 ± 13.26	8.72 ± 15.11	8.34 ± 5.56	11.79 ± 6.52	<0.001	0.144
**Readmission**	75 (4.1%)	47 (3.5%)	21 (5.0%)	7 (10.5%)	0.018	0.387
**Adjuvant chemotherapy**						
Yes	1332 (73.1%)	901 (67.7%)	378 (89.2%)	53 (79.1%)	<0.001	0.609
No	490 (26.9%)	430 (32.3%)	46 (10.8%)	14 (20.9%)		
**Recurrence**	316 (17.3%)	169 (12.7%)	127 (30.0%)	20 (29.9%)	<0.001	>0.999
Systemic	267 (84.5%)	144 (85.2%)	109 (85.8%)	14 (70.0%)	<0.001	>0.999
Local	32 (10.1%)	18 (10.7%)	9 (7.1%)	4 (20.0%)		
Combined	18 (5.6%)	7 (4.1%)	9 (7.1%)	2 (10.0%)		

WD, well-differentiated; MD, moderately differentiated; PD, poorly differentiated; SD, standard deviation; MSI-H, microsatellite instability-high; CDC, Clavien-Dindo classification.

### Overall Survival (OS)

**Figure 1:**
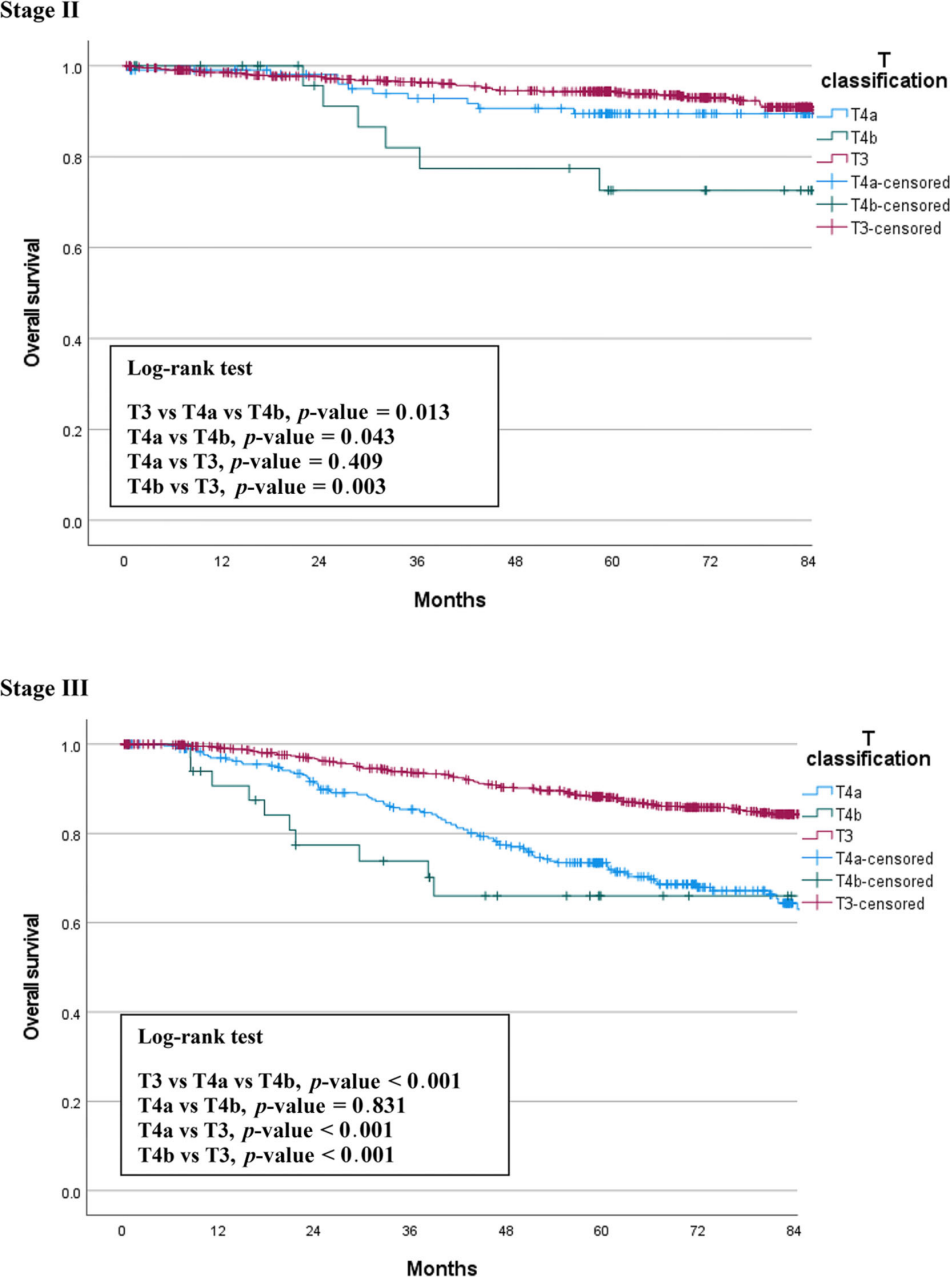
Overall survival of T3 vs. T4 patients by stage.

We compared OS among the T3, T4a, and T4b groups by stage. The median follow-up period was 66 months (0.5 – 128). In patients with stage II cancer, the 5-year OS rates were 93.8% for T3, 89.5% for T4a, and 72.6% for T4b patients. This difference was statistically significant (p = 0.013), and there was also a significant difference between the T4a and T4b groups (p = 0.043). In stage III patients, the 5-year OS rates were 88.1% for T3, 72.4% for T4a, and 66.0% for T4b patients. This difference was also statistically significant (p < 0.001), but there was no significant difference between the T4a and T4b groups (p = 0.831). **Figure 1** shows the OS curves of the three groups.

In multivariable Cox regression analyses, cell type, lymphatic invasion, and tumor budding were excluded from the final models to avoid multicollinearity. Age less than 60 (HR 0.39, 95% CI 0.20-0.74, p = 0.004) and adjuvant chemotherapy (HR 0.32, 95% CI 0.16-0.61, p = 0.001) were independent factors associated with improvements in OS, while cancer perforation (HR 5.00, 95% CI 1.51-16.52, p = 0.008) and perineural invasion (HR 1.93, 95% CI 1.01-3.67, p = 0.045) were independent risk factors. T classification was not a significant independent risk factor (T3, reference; T4a, HR 1.20, 95% CI 0.53-9.58, p > 0.999; T4b, HR 2.26, 95% CI 0.53-9.58, p = 0.532) (**Table 3**).

**Table 3:** Univariable and multivariable cox regression analysis of overall survival.

	Reference		Univariable analysis			Multivariable analysis		
			HR	95% CI	p-value	HR	95% CI	p-value
**T classification**	T3	T4a	2.5	1.82-3.43	<0.001	1.20	0.53-2.68	>0.999
		T4b	3.21	1.70-6.03	<0.001	2.26	0.53-9.58	0.532
**Age**	≥60	<60	0.31	0.23-0.41	<0.001	0.39	0.20-0.74	0.004
**Sex**	Female	Male	1.06	0.82-1.35	0.670			
**Preoperative CEA**	<5	≥5	1.58	1.22-2.04	<0.001	1.61	0.92-2.80	0.094
**Preoperative CA19-9**	<37	≥37	1.42	1.05-1.90	0.020	1.74	0.92-3.27	0.087
**Cancer obstruction**	N	Y	2.09	1.61-2.69	<0.001	0.55	0.28-1.05	0.070
**Cancer perforation**	N	Y	3.25	1.44-7.30	0.004	5.00	1.51-16.52	0.008
**Node positivity**	+	–	0.43	0.32-0.57	<0.001	0.55	0.3-1.01	0.055
**Cell differentiation**	PD	WD	0.38	0.22-0.64	<0.001			
		MD	0.55	0.36-0.85	0.007			
**Lymphatic invasion**	N	Y	1.76	1.38-2.25	<0.001			
**Perineural invasion**	N	Y	1.76	1.37-2.24	<0.001	1.93	1.01-3.67	0.045
**Vascular invasion**	N	Y	2.03	1.56-2.65	<0.001			
**Tumor budding**	N	Y	1.69	1.29-2.21	<0.001			
**MSI-H**	N	Y	0.74	0.41-1.32	0.306			
**Morbidity (CDC grade ≥3)**	N	Y	2.23	1.43-3.45	<0.001	1.87	0.98-3.55	0.058
**Adjuvant chemotherapy**	N	Y	0.44	0.34-0.56	<0.001	0.32	0.16-0.61	0.001

CEA, carcinoembryonic antigen; CA 19-9, carbohydrate antigen 19-9; MSI-H, microsatellite instability-high; CDC, Clavien-Dindo classification.

### Cancer-Specific Survival (CSS)

**Figure 2:**
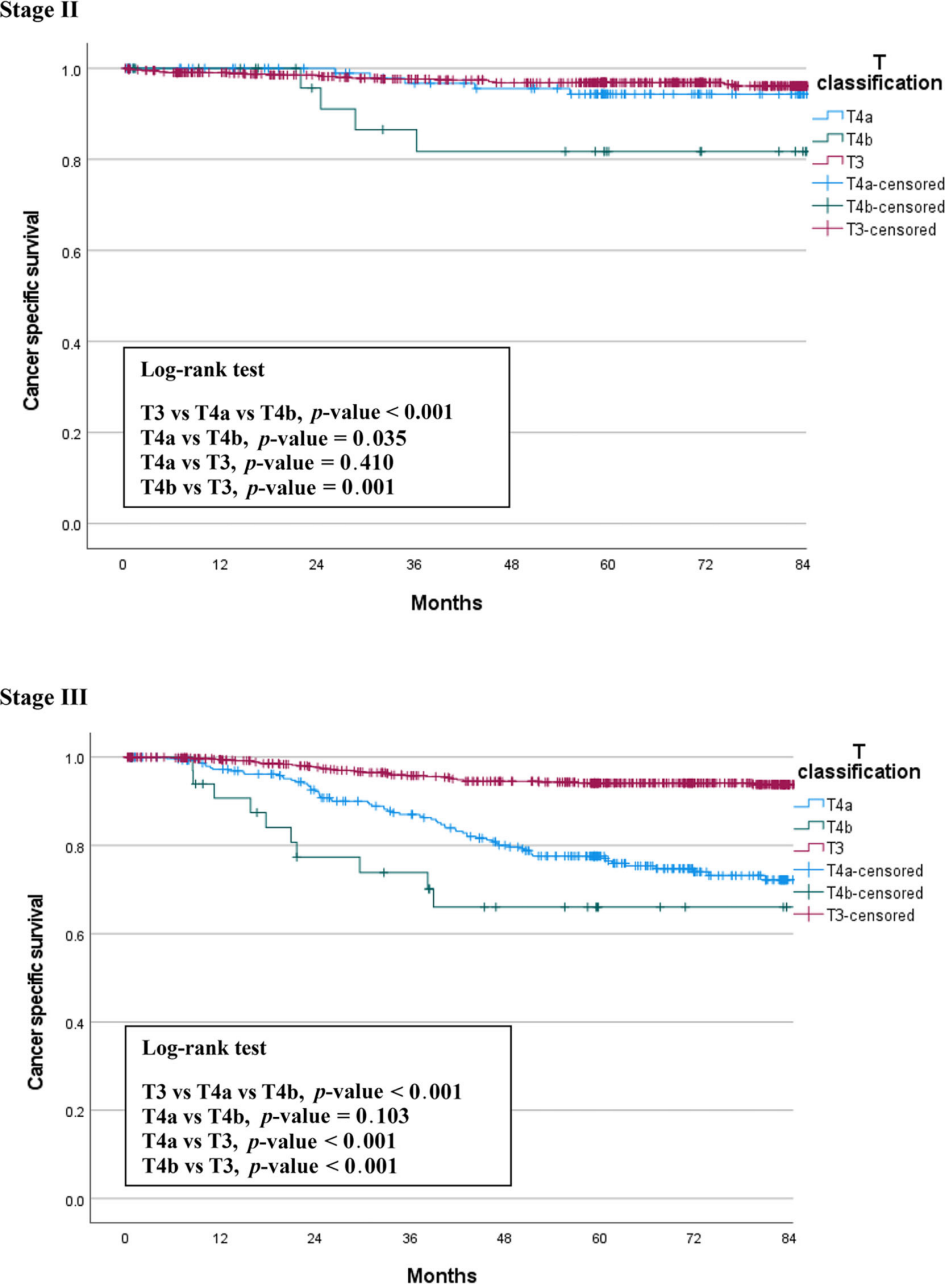
Cancer-specific survival of T3 vs. T4 patients by stage.

We also compared CSS among the three groups by stage. **Figure 2** shows the CSS curves of the three groups, which are similar to the OS curves. In stage II patients, the 5-year CSS rates were 96.9% for T3, 94.4% for T4a, and 81.7% for T4b patients. This difference between the three groups was statistically significant (p < 0.001). The difference between T4a and T4b was also significant (p = 0.035). In stage III patients, the 5-year CSS survival rates were 94.2% for T3, 77.1% for T4a, and 66.0% for T4b patients. This difference between the three groups was statistically significant (p < 0.001), but there was no significant difference between the T4a and T4b groups (p = 0.103) (**Figure 2**).

In multivariable Cox regression analyses, preoperative CEA, cancer obstruction, node positivity, lymphovascular invasion, perineural invasion, tumor budding, morbidity, and adjuvant chemotherapy status were excluded from the final models to avoid multicollinearity. age less than 60 (HR 0.61, 95% CI 0.40-0.92, p = 0.020) and well differentiated tumors (poorly differentiated, reference; well differentiated, HR 0.23, 95% CI 0.09-0.54, p = 0.001; moderately differentiated, HR 0.41, 95% CI 0.23-0.70, p = 0.001) were independent factors for improved CSS, while elevated preoperative CA 19-9 level (HR 1.70, 95% CI 1.10-2.59, p = 0.015) and cancer perforation (HR 4.41, 95% CI 1.72-11.29, p = 0.002) were independent risk factors. Unlike overall survival, T classification was independently associated with CSS; T4b showed relatively higher HR than T4a, when respectively compared to T3 (T3, reference; T4a, HR 2.27, 95% CI 1.37-3.72, p < 0.001; T4b, HR 3.53, 95% CI 1.53-8.08, p < 0.001) (**Table 4**).

**Table 4:** Univariable and multivariable cox regression analysis of cancer-specific survival.

			Univariable analysis			Multivariable analysis		
	Reference		HR	95% CI	p-value	HR	95% CI	p-value
**T classification**	T3	T4a	4.51	2.95-6.90	<0.001	2.27	1.37-3.72	<0.001
		T4b	6.72	3.28-13.76	<0.001	3.53	1.53-8.08	<0.001
**Age**	≥60	<60	0.41	0.28-0.58	<0.001	0.61	0.40-0.92	0.020
**Sex**	Female	Male	0.97	0.69-1.34	0.841			
**Preoperative CEA**	<5	≥5	1.60	1.14-2.25	0.006			
**Preoperative CA19-9**	<37	≥37	1.57	1.07-2.27	0.019	1.70	1.10-2.59	0.015
**Cancer obstruction**	N	Y	2.60	1.86-3.62	<0.001			
**Cancer perforation**	N	Y	4.72	1.93-11.52	<0.001	4.41	1.72-11.29	0.002
**Node positivity**	+	–	0.32	0.21-0.48	<0.001			
**Cell differentiation**	PD	WD	0.19	0.09-0.39	<0.001	0.23	0.09-0.54	0.001
		MD	0.41	0.25-0.67	<0.001	0.41	0.23-0.70	0.001
**Lymphatic invasion**	N	Y	2.56	1.82-3.59	<0.001			
**Perineural invasion**	N	Y	2.66	1.91-3.77	<0.001			
**Vascular invasion**	N	Y	2.69	1.92-3.77	<0.001			
**Tumor budding**	N	Y	2.14	1.46-3.13	<0.001			
**MSI-H**	N	Y	0.64	0.28-1.46	0.291			
**Morbidity (CDC grade ≥3)**	N	Y	2.58	1.49-1.49	<0.001			
**Adjuvant chemotherapy**	N	Y	0.14	0.05-0.38	<0.001			

CEA, carcinoembryonic antigen; CA 19-9, carbohydrate antigen 19-9; WD, well-differentiated; MD, moderately differentiated; PD, poorly differentiated; MSI-H, microsatellite instability-high; CDC, Clavien-Dindo classification.

## Discussion

The 1st AJCC guidelines were released in 1977; they have been updated several times over the years, and the 8th edition was published in 2016 (17, 18). There are several changes in the new edition, chief among which is that peritoneal metastasis is included in the new staging system as stage IV C (17, 19).

T4 was first subdivided into T4a and T4b in 2000 (20, 21). This change was incorporated into the 6th edition of the TNM supplement (21, 22) and the College of American Pathologists (CAP)-approved protocol for specimen examination of primary CRC patients (21, 23). The T4a designation indicated that the tumor directly invades other organs or structures, while T4b indicated that the tumor penetrates the visceral peritoneum (20, 21, 23). However, that was inverted in the 7th edition of the AJCC guidelines to the present meaning, in which a T4a tumor only penetrates the visceral peritoneum and a T4b tumor directly invades or is adherent to other organs or structures (2). According to two studies by Gunderson et al., who analyzed 35,829 rectal cancer and 109,953 colon cancer patients documented in the Surveillance, Epidemiology, and End Results (SEER) population-based dataset, T4a was associated with a more favorable prognosis than T4b for each N classification of both colon and rectal cancer (4, 5). Contrary to these results, a study published in 2019 by Baguena et al. which compared pathologically confirmed T3, T4a, and T4b showed that T4a was an independent risk factor for local recurrence, peritoneal carcinomatosis, worse disease-free survival, and cancer-specific survival, and they suggested that current AJCC classification should be reconsidered (8).

In the present study, we compared three groups of patients with each other to understand the relationship between T4a and T4b based on T3. We found the same trends in the differences between the survival curves of OS and CSS (**Figures 1**, **2**). T4a and T3 showed similar survival rates over time in stage II colorectal cancer patients, but T4b was associated with a significant reduction in survival rate. On the contrary, in stage III patients, T4a and T4b showed similar survival rates over time, while T3 showed significantly higher survival rates. These results mean that, for long-term outcomes, in the absence of lymph node metastases, invading other organs or structures is an important factor for prognosis. However, if there are lymph node metastases, the serosal invasion itself, whether T4a or T4b, is more important for prognosis than invasion of other organs. Moreover, in the multivariable Cox analysis, depth of invasion was an independent prognostic factor for CSS; the hazard ratio of T4a and T4b were 2.27 and 3.53, respectively, compared to T3. These results support the current conviction that T4b is associated with poorer prognosis than T4a.

We also found that more T4b than T4a patients showed elevated CA 19-9 levels, and that this was an independent risk factor for decreased CSS. Many studies have reported that preoperative elevations in CA 19-9 levels predict poor survival (24–26). Our study showed that elevated preoperative CA19-9 was more indicative of a poor prognosis than elevated preoperative CEA in patients with a tumor invasion depth of T3 or higher. This may indicate that CA 19-9 testing, along with CEA, is a vital part of the preoperative workup, especially for patients with tumor invasion of the muscularis propria and deeper structures. In addition, there were more MSI-H colon cancer patients in the T4b group than in theT4a group. Many studies have shown that MSI-H colon cancer has a better prognosis than MSI-low or microsatellite-stable (MSS) colon cancer (27–30). However, MSI-H was not identified as an independent prognostic factor in our study. The higher proportion of MSI-H in T4b, which had a poorer prognosis than in T4a, may indicate that MSI status did not have a significant impact on prognosis in T4b, but MSI-H may have been overestimated in T4b due to the small number of T4b patients. Therefore, further studies with larger numbers are needed.

This study has several limitations. First, this is a retrospective study performed in a single center. Second, it included only a small number of T4b colorectal cancer patients, and it may not reflect the true impact of T4b tumor properly. Third, according to the current NCCN guidelines, neoadjuvant chemotherapy is recommended if T4b is clinically suspected. However, this recommendation has been included in the NCCN guideline since 2016 (31), so the cases from 2010 to 2014 that included patients in this study were not applicable. Therefore, during this period in our hospital, surgery was performed first if the tumor was completely resectable. And then, if T4b was confirmed in the postoperative pathological examination, adjuvant chemotherapy was recommended. Thus, the survival of T4b patients who underwent chemotherapy before surgery according to the current guidelines was not studied. Fourth, genetic tests such as KRAS, NRAS, and BRAF related to prognosis were not performed in all patients, so this could not be analyzed. Fifth, we did not analyze the recurrence or peritoneal seeding according to T4a/b classification, which is also an important oncologic outcome. However, this study also has several strengths stemming from its single-center setting which, for example, enabled precise data collection, use of similar treatment protocols in most patients, and the fact that the same multidisciplinary team operated on all of the patients.

In conclusion, T4a was associated with greater OS and CSS rates than T4b, especially in stage II patients. Moreover, T classification was an independent risk factor in CSS, with a higher hazard ratio seen in T4b than T4a compared to T3 patients. The findings of the present study support the current AJCC guideline that T4b tumors are at a more advanced stage than T4a tumors, and that preoperative CA 19-9 measurements are essential for predicting prognosis. A multicenter study with a larger sample size and more data on the relationship between recurrence and T4a/T4b staging is needed.

## Data Availability Statement

The raw data supporting the conclusions of this article will be made available by the authors, without undue reservation.

## Ethics Statement

The studies involving human participants were reviewed and approved by Institutional Review Board of Samsung Medical Center. Written informed consent for participation was not required for this study in accordance with the national legislation and the institutional requirements.

## Author Contributions

JL and JH contributed to conception and design of the study. JL, JH, WL, SY, HK, YC, YP, and JS organized the database. JL performed the statistical analysis. JL and JH wrote the first draft of the manuscript. All authors contributed to manuscript revision, read, and approved the submitted version.

## Conflict of Interest

The authors declare that the research was conducted in the absence of any commercial or financial relationships that could be construed as a potential conflict of interest.

## Publisher’s Note

All claims expressed in this article are solely those of the authors and do not necessarily represent those of their affiliated organizations, or those of the publisher, the editors and the reviewers. Any product that may be evaluated in this article, or claim that may be made by its manufacturer, is not guaranteed or endorsed by the publisher.
